# Ag(I) Biosorption and Green Synthesis of Silver/Silver Chloride Nanoparticles by *Rhodotorula mucilaginosa* 1S1

**DOI:** 10.3390/nano13020295

**Published:** 2023-01-11

**Authors:** Antonio J. Muñoz, Francisco Espínola, Encarnación Ruiz, Manuel Moya, Eulogio Castro

**Affiliations:** 1 Department of Chemical, Environmental and Materials Engineering, Universidad de Jaén, Campus Las Lagunillas, 23071 Jaén, Spain; 2 Centre for Advanced Studies in Earth Sciences, Energy and Environment (CEACTEMA), Universidad de Jaén, Campus Las Lagunillas, 23071 Jaén, Spain

**Keywords:** bioremediation, yeast, heavy metals, Ag/AgCl-NPs, XRD, FT-IR

## Abstract

The efficiency of *Rhodotorula mucilaginosa* 1S1 as an Ag(I) biosorbent and at the same time its ability to biosynthesize recoverable silver nanoparticles is evaluated. Kinetic, equilibrium and thermodynamic tests are carried out for 19 °C, 27 °C and 37 °C, from which the process is adjusted to a pseudo second-order kinetics and to the Freundlich model, while optimal operational conditions are determined at 27 °C. The thermodynamic study shows positive values for enthalpy (ΔH: 133.23 kJ/mol) and entropy (ΔS: 0.4976 kJ/(mol K)), while the Gibbs free energy (ΔG) value is 12.136 kJ/mol. For a metal concentration of 459 mg/L, a maximum biosorption capacity (q_m_) of 137.2 mg/g at 19 °C is obtained, while for 100 mg/L concentration a q_m_ value of 60.44 mg/g is obtained at the same temperature. The mechanisms involved in the biosorption process are studied by infrared spectroscopy, X-ray diffraction and scanning and transmission electron microscopy, while the nanoparticle synthesis is evaluated by ultraviolet–visible spectrophotometry (UV-vis) and transmission electron microscopy. The results indicate that the biomass is a good biosorbent and also has the ability to synthesize silver nanoparticles (Ag/AgCl) with sizes between 12 nm and 20 nm.

## 1. Introduction

The strong presence of heavy metals in the natural environment because of human activity is one of the greatest challenges for the development of environmental purification techniques. Heavy metals have wide applications in industry and therefore their presence in waste streams that end up in natural ecosystems is common [1,2]. One of the Sustainable Development Goals (SDG) of the United Nations to be achieved in 2030 proposes to improve water quality, reduce pollution, eliminate discharges and minimize the release chemicals and hazardous materials, to halve the percentage of untreated wastewater and substantially increase recycling and safe reuse of water. This objective makes perfect sense when it is known that 80% of the wastewater flows back into the ecosystem without being treated or reused, to which must be added the problems derived from the direct lack of water and the contamination of the soils of the cultivation and aquifers where it is not treated.

Recent studies have focused on environmental contamination by silver ions [3,4]. Silver is a rare element in the Earth’s crust but can be found in various oxidation states, mainly as metallic silver, Ag(0), or as a cation, Ag(I). In surface waters, it can appear in the ionic or precipitated state forming sulfides, bicarbonates or sulfates; it can also do so as silver chloride, AgCl, or any of its species. In fresh water, it can appear dissolved as Ag(I) or associated with colloidal organic macromolecules that form different chlorocomplexes when salinity changes. It is a metal with no known biological function that also reaches the water as a result of its use in various branches of industry: medicine, electricity, electronics, catalysts, alloys, welding, computing, etc. [5]. Once in the environment, it can precipitate in sediments where pH conditions can return it to its ionic state, in which it is much more available and has high short- and long-term potential toxicity to fish, invertebrates, algae, cyanobacteria, and microorganisms in general [3].

Silver also has the capacity to bioaccumulate and biomagnify when passing through the food chain [6]. Its powerful negative effect on the environment is a consequence of its high toxicity to living cells, its free ionic form being one of the most toxic metal species known to aquatic organisms (fish and phytoplankton), invertebrates in general, and bacteria. Silver can inhibit the functioning of essential enzymes in cell metabolism by binding to their sulfhydryl groups and also competes with selenium, vitamin E and copper. In its form of silver nitrate, AgNO_3_, it is lethal to fish at very low concentrations: 5–70 μg/L [7,8,9].

Although effective physicochemical techniques have been implemented in recent decades to remove heavy metals from contaminated effluents, in many cases these metals are found in very low concentrations (<100 mg/L), making their application useless and costly. For this reason, a cheap, ecological and highly effective technology at low concentrations called biosorption began to be developed. Biosorption takes advantage of the functional groups present in residual biomasses and the microbial metabolism itself, in the case of active biomasses, to retain dissolved metal ions [10]. An additional advantage of heavy metal biosorption is that some of the mechanisms involved can promote the alteration of the chemical nature of the metal, which is then precipitated as metallic nanoparticles [11]. This particularity, in turn, offers great potential possibilities in nanotechnology and nanomedicine by offering as an added value to the biosorption process the obtaining of metallic nanoparticles that can be recovered and used in other technologies [12].

The main aim of this work was to study the biosorption capacity of Ag(I) ions by a ubiquitous yeast isolated from urban wastewater and to perform a thermodynamic study of the process. It was also sought to obtain silver nanoparticles (Ag-NPs) as an added value to the biosorption process. The yeast *Rhodotorula mucilaginosa* 1S1 had been isolated and molecularly identified in a previous work [13] from wastewater from the province of Jaén (Spain). In preliminary tests, it had shown good potential conditions for the biosorption of Pb, Zn and Ag [14].

## 2. Materials and Methods 

### 2.1. Preparation of the Biomass, the Cell Extract and Biosorption Tests

*Rhodotorula mucilaginosa* 1S1 had been kept in glycerol at −20 °C and, once activated for use, it was maintained by weekly reseeding and stored at 5 °C. To obtain the biomass, an orbital SI-600R (Lab Companion, Warpsgrove Ln, Chalgrove, Oxfordshire, OX44 7XZ, UK) was used. Using a pre-inoculum of 24 h of growth at 27 °C and 150 rpm, the microorganism was grown in YPG medium (yeast, 10 g/L; peptone, 10 g/L; glucose, 20 g/L). Identical conditions were reproduced in 6–250 mL flasks with 200 mL of this medium until reaching the exponential phase of growth (established in 12 h from a previously obtained growth curve). Next, the biomass was centrifuged at 6000 rpm and washed with an electrolyte solution of 0.1 M HNO_3_ and the final pH of the culture was adjusted to 5 with 0.1 M NaOH until all traces of YPG medium were removed. From this cell suspension, the dry weight was calculated. Once the optimal operating conditions were known, after the experimental design, the biomass was washed with an HNO_3_ solution adjusted to the optimal pH. The biosorption tests were done in duplicate at 27 °C and 200 rpm in the same orbital described above and in 100 mL flasks with a volume of Ag(I) solution of 50 mL. Samples were filtered (0.22 µm filter) and analyzed by atomic absorption spectroscopy (AAS) on a Perkin Elmer AAnalyst 800 instrument (Midland, ON, Canada). The biosorption capacity (q) was defined as the milligrams of metal retained per gram of dry biomass (mg/g) and was calculated from Equation (1):

(1) $$q=\frac{(C_{i}-C_{f})V}{m}$$

where C_i_ and C_f_ are the initial and final concentration of Ag(I) in mg/L, respectively; V is the volume of metal solution (0.05 L) and m is the dry mass of biomass (g).

To obtain the cell extract of *Rhodotorula mucilaginosa* 1S1 from a 24 h pre-inoculum, 10 flasks of 250 mL with 200 mL of YPG under the same conditions as above were placed. After 48 h, the flasks were kept at 5 °C for 72 h and the biomass produced was centrifuged and washed as described above until a concentrated cell suspension was obtained. In flasks with a volume of 250 mL, 5 g of fresh biomass were put in contact with 100 mL of sterile ultrapure water and kept stirring for 4 days (150 rpm) at 27 °C. The suspension was then filtered (PVDF/45 µm filters) to obtain the cell extract.

### 2.2. Experimental Design

All the tests were carried out under the conditions described in the previous section starting from the cell suspension and with a metallic solution of Ag(I) with a concentration of 100 mg/L prepared with the same electrolytic solution used to wash the biomass. This Ag(I) solution was prepared by dilution starting from a 1 g/L stock solution that had been made with AgNO_3_ over 0.1 M HNO_3_ electrolyte. The assay was maintained for 4 days to ensure that the biosorption process was complete. Table S1 shows the experimental conditions obtained from a central rotatable design composed of five central points and in which two factors were studied: pH (A) and dose of biosorbent (B). As a dependent variable, the biosorption capacity in the equilibrium (q_e_, mg/g), obtained from Equation (1) was considered. For the analysis of results, the Design-Expert^®^ software version 8.0.7.1 (Stat-Ease, Inc., Minneapolis, MN, USA) was used, which also allowed the obtaining of the analysis of variance (ANOVA).

### 2.3. Silver Biosorption Kinetics

To study the adsorption kinetics of silver ions by *Rhodotorula mucilaginosa*, experiments were carried out starting from a fixed volume of solution (V) and a fixed initial concentration of silver (C_i_). The yeast was added in a predetermined quantity (m), and samples of the solution were taken at different times until we considered that the biomass had become saturated and did not have a greater adsorption capacity.

After analyzing the dissolution samples and determining the silver content at different times (C_t_), the amount of metal adsorbed by the biomass can be determined by Equation (1), substituting C_f_ for C_t_ and q for q_t_, where q_t_ represents the silver adsorbed by the biomass at time t.

For a same adsorbent–adsorbate system, the comparison of experimental kinetic data with different initial values can lead to erroneous conclusions. Whenever attempting to compare kinetic data from different experiments, it must be confirmed that the system is the same and that the initial adsorbent and adsorbate concentrations are the same.

To transform the experimental data and refer them to the same initial values, the proportionality factor (f_p_) is defined as the ratio between the target biomass (m_o_) and the target metal (C_o_V_o_) (initial reference metal mass) divided by the ratio between the experimental biomass (m_r_) and metal (C_r_V_r_) (actual initial mass of metal for each experiment).

(2) $$f_{p}=\frac{m_{o}}{m_{r}}\frac{C_{r}V_{r}}{C_{o}V_{o}}$$

Pseudo first-order (Lagergren’s model), pseudo second-order (Ho’s model), Elovich and intra-particle diffusion models have been applied for the kinetic study of the experimental data [15].

Lagergren’s model assumes that adsorption conforms to a chemical reaction of order 1. Equation (3) shows the velocity equation and its integrated form for the boundary conditions: t = 0 q = 0 y t = t q = q_t_:

(3) $$\frac{dq}{dt}=k_{1}(q_{e}-q)\;\to \;q_{t}=q_{e}\left(1-e^{-k_{1}t}\right)$$

where q_e_ (mg/g) is the equilibrium adsorption capacity and k_1_ (min^−1^) is the kinetic rate constant. Ho’s model assumes that adsorption fits a second-order reaction rate equation. Equation (4) shows the rate equation and its integrated form for the same boundary conditions as in the previous case:

(4) $$\frac{dq}{dt}=k_{2}{\left(q_{e}-q\right)}^{2}\;\to \;q_{t}=\frac{q_{e}}{1/(q_{e}k_{2}t)+1}$$

where k_2_ (g/(min mg)) is the kinetic constant.

The Elovich model is an empirical model widely used in chemisorption. Equation (5) represents its kinetic equation and its integrated form:

(5) $$\frac{dq}{dt}=q_{0}e^{-bq}\;\to \;q_{t}=\frac{Ln\;(q_{0}bt+1)}{b}$$

where q_0_ is the initial metal retention rate and b (g/mg) is the Elovich rate constant.

In the intraparticle diffusion model (Equation (6)):

(6) $$q_{t}=k_{p}\sqrt{t}+C$$

where k_p_ is the intraparticle velocity constant and C is a constant indicating the influence of the boundary layer.

### 2.4. Biosorption Equilibrium Study

Equilibrium tests were performed as described in the first paragraph of the Materials and Methods section and at different metal concentrations (30–500 mg/L).

Many isotherm models are available to fit equilibrium data, from single-parameter models such as Henry’s to five-parameter models such as Fritz–Schlunder’s [16]. In this work, the most common two-parameter isotherms such as Langmuir, Freundlich, Temkin and Elovich were used. For three parameters, the Sips and Redlich–Peterson isotherms, both in their different forms of presentation, and the Langmuir–Freundlich isotherm were used.

### 2.5. Temperature and Thermodynamics Study

There are different procedures in the literature to determine the thermodynamic parameters of the adsorption process [17,18,19]. In this work, four different procedures were used. First, the distribution constant (L/g) was used; Equation (7),

(7) $${K_{d}}^{\prime }=\frac{q_{e}}{C_{e}}$$

defined it as the ratio between the adsorption capacity of the biomass and the concentration of adsorbate in the solution at equilibrium. The value of $\left({K_{d}}^{\prime }\right)$ to be used is obtained by plotting $\left(\mathrm{Ln}\;{K_{d}}^{\prime }\right)$ vs. q_e_ and extrapolating to zero [20,21,22]. Second, the same distribution coefficient was used, transformed into dimensionless by multiplying it by the biomass concentration [18], as in Equation (8):

(8) $$K_{d}=\frac{q_{e}}{C_{e}}\frac{m}{V}$$

which extrapolates to zero $\left(\mathrm{Ln}\;K_{d}\right)$ vs. q_e_. Third, the Freundlich constant, modified according to Equation (9), was used [19,23,24].

(9) Kc=KFρ(1000ρ)(1−1n)

where K_c_ is the thermodynamic equilibrium constant and ρ is the density of water (g/cm^3^) at the working temperature. Fourth, the parameter n of the Freundlich isotherm was used [25,26] to determine the Gibbs free energy (ΔG=−nRT) and, with this, the enthalpy and entropy variation. The Gibbs free energy is given by the expression $\left(\Delta G=-RT\cdot Ln\;K_{c}\right)$ [17] as a function of the thermodynamic equilibrium constant (K_c_). This, in turn, is a function of enthalpy, entropy and temperature according to the expression (ΔG=ΔH−TΔS). Equating both expressions gives the van’t Hoff equation, which relates the equilibrium constant to the temperature (Equation (10)):

(10) $$Ln\;K_{c}=-\frac{\Delta H}{R}\frac{1}{T}+\frac{\Delta S}{R}$$

### 2.6. Determination of Biosorption Mechanisms

Biomass samples were collected before and after the biosorption tests, then they were washed and lyophilized to finally proceed to their analysis using Fourier transform infrared spectroscopy (FT-IR) in a VERTEX 70 spectrometer (Bruker Corporation, Billerica, MA, USA) by total attenuated reflection (ATR) in the range of 4000–400 cm^−1^. The objective was to identify the participation in the biosorption of Ag(I) of the functional groups present in the biomass.

To identify the presence of crystalline structures compatible with the existence of reduction processes during silver biosorption, the X-ray diffraction (XRD) technique was used. An Empyrean model instrument from Malvern Panalytical (Malvern, WR14 1XZ, UK) was used. The equipment has a copper radiation source with an emission wavelength (λ) of 1.5406 Å and the measurements were performed continuously using a K-Alpha2/K-Alpha1 wavelength ratio of 0.5, a scan range for angle 2θ from 10° to 90° with a step size of 0.013, a voltage of 45 V and an exposure time of 20 min. To calculate the size of the crystals, the Debye–Scherrer equation (Equation (11)) was applied:

(11) $$d=\frac{k\lambda }{\beta \cos\theta }$$

where d is the mean diameter (nm) of the crystals, k is Scherrer’s constant, which takes a value of 0.9, λ is the wavelength (nm) of the incident X-rays, β is the width of the mean XRD peak height expressed in radians and θ is the Bragg diffraction angle.

To study cell topography and visually and spectrally identify the presence of silver precipitates on the biomass surface, scanning electron microscopy coupled to an X-ray scattering detector (FESEM-EDX) was applied. MERLIN equipment from Carl Zeiss (Göttingen, Germany) was used. At the same time, to identify the bioaccumulation mechanisms that incorporate the metal into the microbial cytoplasm, a FEI-TITA-G2 transmission electron microscope (TEM) was used. In both cases, the samples were collected before and after biosorption and for scanning microscopy they were prepared as described in Muñoz et al. [27], while for transmission microscopy, an epoxy resin insertion stage was added to the previous process.

### 2.7. Synthesis of Silver Nanoparticles

To synthesize Ag-NPs, the cell extract previously obtained with a 10 mM Ag(I) solution was contacted. The metal solution was prepared on the same electrolytic solution of the extract. The synthesis was carried out in triplicate in 250 mL opaque flasks in which 90 mL of extract and 10 mL of metallic solution were added to obtain a final concentration of 1 mM Ag(I). A control was also prepared that only contained the matrix of both solutions, to correct the baseline. The flasks were kept at 27 °C under agitation (150 rpm) and daily measurements were made by UV-vis spectrophotometry until the spectra remained constant, indicating that the synthesis had been completed. The samples were measured in the range of 200 to 700 nm in a UV-Vis Shimadzu UV-1800 spectrometer (Römerstrasse 3, Reinach BL, Switzerland). The resulting solutions were washed and centrifuged at 11,000 rpm to obtain a colloidal suspension of nanoparticles. Finally, to morphologically analyze the Ag-NPs recovered in the synthesis assays, a transmission electron microscope (TEM) JEOL JEM- 1010 (11 Dearborn Road, Peabody, MA, USA) was used. Statistical analysis of size distribution was applied to these images using ImageJ software.

## 3. Results and Discussion

### 3.1. Experimental Design: Optimization of the Factors Involved

Table S1 shows the results obtained from the experimental design tests, showing biosorption capacity values between 29.07 and 69.75 mg/g. The analysis of the data using the Design-Expert^®^ software resulted in Equation (12):

(12) $$q_{e}=56.26+29.44A-346.80B-12.91AB-1.27A^{2}+313.30B^{2}\pm 0.26$$

**Figure 1:**
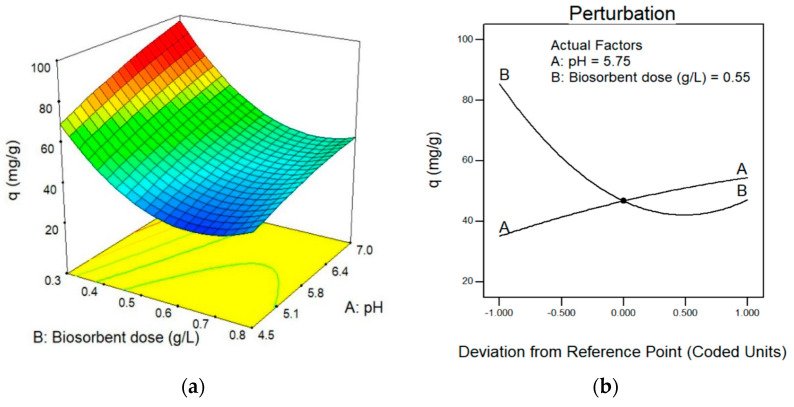
(**a**): Response surface plot for silver biosorption by *Rhodotorula mucilaginosa* 1S1 at 27 °C and initial silver concentration of 100 mg/L. Effect of the factors studied on the biosorption capacity (q_e_). (**b**): Perturbation plot showing the effect of pH (A) and dose of biosorbent (B) on the equilibrium biosorption capacity of silver (q_e_).

Equation (12), of the quadratic model, is significant (*p*-value < 0.0001); it has no lack of fit (*p*-value = 0.7935), a coefficient of determination of 0.9997, a standard deviation of 0.26 and a coefficient of variation of 0.53%. The model determined an optimal pH of 7 and an optimal biosorbent dose of 0.30 g/L. The graph in Figure 1a shows that high pH values and low biosorbent dose values favor the biosorption process. At the same time, it was shown that the dose of biosorbent is the factor that most influences the ability to biosorb silver (Figure 1b).

### 3.2. Biosorption Kinetics

Table S2 shows the experimental results obtained from the initial data at 19, 27 and 37 °C, respectively. The calculated f_p_ values (Equation (2)) (0.9434, 0.9981 and 0.9965, respectively, at each temperature) were applied to the data in Table S2, and the discussion will be carried out as if initially all experiments were started with 0.3 g/L biomass and 100 mg/L Ag(I). These results were fitted to the kinetic models by nonlinear regression applying the Levenberg–Marquardt algorithm. First, the results were adjusted for temperature, which allowed the determination of the two kinetic models that best fit the experimental data. Among all the models applied, the intraparticle diffusion model provides the worst statistics (R^2^ < 0.4 at all three temperatures), followed by the Elovich model (R^2^ ≈ 0.6). This is probably because the system of a living microorganism does not resemble a porous particle and the Elovich equation is more specific to chemisorption than to biosorption.

The coefficients of determination obtained by applying the Lagergren model were 0.86, 0.82 and 0.95 at temperatures of 19, 27 and 37 °C, respectively. For the Ho model, R^2^ = 0.97, 0.92 and 0.98, respectively. This statistic indicates, a priori and individually by temperature, that the adsorption kinetic data fit best to a pseudo second-order reaction and somewhat worse to a pseudo first-order reaction. Both models will be applied to the dataset for all temperatures. 

The maximum adsorption capacities were plotted versus temperature. Adjustments were made with different types of equations, linear, exponential, potential, etc., with the result that the best fit to the data is a linear-type equation. Therefore, in Equations (3) and (4) q_e_ was replaced by a linear equation of the absolute temperature. The kinetic constant fits the Arrhenius equation. As a result of substituting the above expressions, Equations (13) and (14) are obtained for the pseudo first-order and pseudo second-order kinetics, respectively. The equations were used to fit the adsorption kinetic data. The software used was StatGraphics Centurion version XIX, (Statpoint Technologies, Inc., Warrenton, VA, USA) and the Levenberg–Marquardt algorithm was applied for the nonlinear fit.

(13) $$q_{t}=(a+bT)\left(1-e^{-k_{01}e^{\left(-\;\frac{E_{a}}{RT}\right)}t}\right)\pm \varepsilon$$

(14) $$q_{t}=\frac{a+bT}{1/\left[k_{02}e^{\left(-\;\frac{E_{a}}{RT}\right)}(a+bT)t\right]+1}\pm \varepsilon$$

where a and b are the coefficients of the equation to determine q_e_, k_01_ and k_02_ are the pre-exponential terms of the Arrhenius equation for the kinetic constants k_1_ and k_2_, respectively, R is the gas constant (8.3145 J/(mol K)), E_a_ the activation energy (J/mol) and T the absolute temperature (K). The standard deviation (ε) of the fit model was introduced in the equations as indicative of the dispersion of the model when reproducing the experimental results.

Table 1 shows the results obtained after fitting the experimental data with Equations (13) and (14.) As a result, all models are statistically significant, there is no lack of fit and they have low coefficients of variation. The *p*-value of the coefficients is, in most cases, less than 0.01.

As can be seen in Table 1, the model with the best statistics is the pseudo second-order model, since it has the best coefficient of determination and the lowest relative error. However, the differences with the pseudo first-order model are small, and the statistics are similar, so it could be stated that both models reproduce the adsorption kinetics, although the pseudo second-order model is better. This is consistent with Moussout et al. [28] and Revellame et al. [29] of using more than one statistic to determine that one model is better than another.

**Figure 2:**
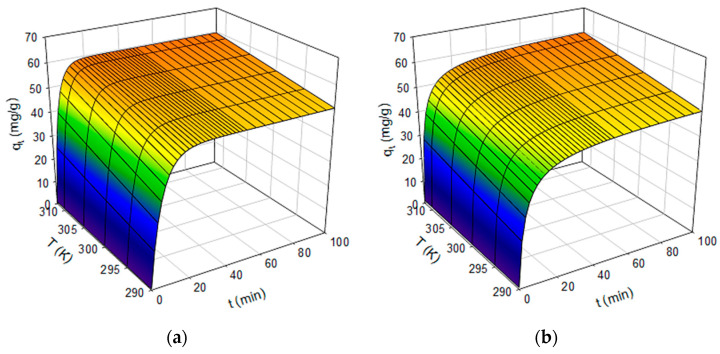
(**a**): Response surface graph for pseudo first-order kinetic model in Ag(I) biosorption by *Rhodotorula mucilaginosa* 1S1. (**b**): The same case for the pseudo second-order kinetic model.

Figure 2a,b show, in three-dimensional format, the response surfaces drawn by both models, showing the great similarity between them. Although if the curvature that they present around 20 min is closely observed, it can be affirmed that the pseudo first-order model predicts that at this moment almost 100% of the maximum adsorption capacity has been reached, while for the pseudo second-order model at 20 min 90% of this capacity is reached. This is very important when designing a continuous adsorption process where residence time is the main design variable. Therefore, it can be stated that it is not convenient to exceed 20 min of continuous treatment to have a relatively profitable silver decontamination operation. The above is consistent with the low values obtained for the activation energy (E_a_ < 40 kJ/mol) that would indicate that there is a predominance of physical adsorption [23,25].

### 3.3. Biosorption Isotherms

Table S3 shows the experimental results of silver adsorption equilibrium by the yeast *R. mucilaginosa*. This table shows that as the metal concentration in the equilibrium increases, the adsorption capacity of the biomass also increases, although this increase is greater at low concentrations than at high concentrations.

Table 2 shows the isotherm models used in the discussion of this work, all of them with two parameters. The isotherms with more than two parameters are not shown because all the models used, seven three-parameter models, have provided R^2^ values similar to those obtained with the two-parameter models (R^2^ ≈ 0.93) and worse *p*-values (*p*-value >> 0.05) in some of the parameters.

In the models of Table 2, q_e_ represents the amount of silver absorbed by the biomass (mg/g), and C_e_ is the concentration of silver (mg/L) in equilibrium with q_e_ [16]. K_L_ is the Langmuir constant (L/mg), and q_m_ is the maximum adsorption capacity (mg/g). K_F_ is the Freundlich constant (mg/g)(L/mg)^1/n^, and n is an experimental parameter. Values of n greater than 2 indicate a high adsorbent–adsorbate interaction and, therefore, adsorption is very effective [30]. K_T_ is the Temkin constant (L/mg) and B_T_ is a parameter related to the heat of adsorption (mg/g). K_E_ is the Elovich constant (L/mg) and q_E_ is the maximum adsorption capacity (mg/g).

Once only, the two-parameter isotherms were selected; for each isotherm model, the different parameters provided by the software were adjusted with the temperature. Different equations were applied, and it was determined that the equations that best fitted the parameters with temperature were of the linear type. Therefore, Table 2 shows the four isotherms in their final form, once each parameter has been replaced by its temperature-dependent expression. These equations are the ones used to fit all the experimental data in Table S3. As a complement, the parameter ε has been included in all isotherms. After applying nonlinear fit with the models in Table 2 to the data in Table S3, the results (coefficients and statistics) shown in Table 2 were obtained. This table also includes the coefficient of variation (CV) or relative error of each model.

Table 2 shows that the Langmuir isotherm has the worst R^2^, although the standard error of the model is not very large. The rest of the coefficients of determination can be considered as good since 0.88 is a good fit for so much experimental data and only four coefficients to determine, apart from the fact that CV is less than 10%. The standard error of the Temkin isotherm is very similar to that of the Freundlich isotherm, but its CV is higher by almost two percentage points. The highest standard error of the four fitting models is found for the Elovich isotherm, but this is due to the fact that it fits C_e_ vs. q_e_. The CV, which is independent of the variable, is also the highest of all and, although R^2^ is second best, the very high CV indicates that the model does not fit the experimental data well. 

Table 2 shows that the Elovich isotherm is not a good model since two of its coefficients would not be significant. The same is true for the Temkin isotherm with one of its coefficients. In contrast, all the Langmuir isotherm coefficients can be considered significant, even if they are close to the limiting value of 0.05. Finally, the model with the best R^2^, the lowest standard error, the lowest CV and where all coefficients are highly significant is the Freundlich isotherm model. Therefore, it can be stated that the adsorption of silver by *R. mucilaginosa* fits this isotherm.

**Figure 3:**
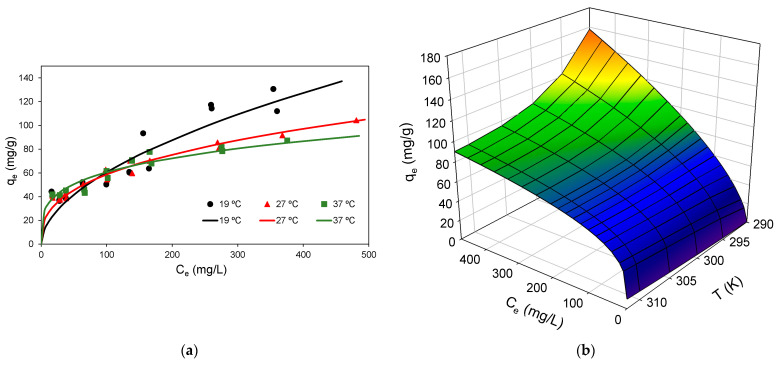
(**a**): Freundlich isotherm for Ag(I) biosorption by *Rhodotorula mucilaginosa* 1S1 at three temperatures and (**b**): response surface graph for the factors studied.

Figure 3a shows the graphical representation of the experimental data and the fit curves at each temperature, obtained with the Freundlich isotherm of Table 2. The response surface drawn by this model is also shown, in the range of the operating variables. Figure 3b shows that for equilibrium concentrations below 100 mg/L the temperature exerts a positive influence on the adsorption capacity, while for higher values it reverses its influence, so that the higher the temperature the lower the adsorption capacity, for the same value of metal concentration at equilibrium. This is due to the value of n increasing with temperature (1.86, 2.75 and 3.86), indicating an increase in the affinity of the adsorbent for the adsorbate, but the desorption rate increases and the biomass is unable to retain such a high amount of metal. Moreover, the Freundlich constant also increases with temperature (5.08, 10.98 and 18.36 mg/g), indicating a high adsorption capacity in very dilute solutions.

Since the Freundlich isotherm model does not give the maximum adsorption capacity, the value of q_e_ can be determined from different values of C_e_. Although in this work the maximum equilibrium concentration reached was 459 mg/L, with a q_e_ of 137.2 mg/g at 19 °C, the isotherm model allows extrapolation to any value of temperature and equilibrium concentration. For C_e_ values of 100 mg/L, a q_e_ of 60.44, 58.65 and 60.53 mg/g was determined at temperatures of 19, 27 and 37 °C, respectively. Thus, assuming a temperature of 19 °C and equilibrium concentrations of 600, 800 and 1000 mg/L Ag(I), q_e_ values of 158.4, 184.9 and 208.5 mg/g, respectively, are determined. The values are much higher than those compiled by Muñoz et al. [14] for different microorganisms and Wang et al. [31] and Liu et al. [32] for nonbiological adsorbents. On the other hand, if a temperature lower than 19 °C is assumed, adsorption will be slower, but the adsorption capacity will increase. Thus, for 15 °C and an equilibrium concentration of 459 mg/L, the calculated q_e_ is 161.9 mg/g, which compared to literature values is quite high. For all these reasons, this biomass is a very good adsorbent of silver ions.

### 3.4. Thermodynamics of the Biosorption Process

Table 3 shows the thermodynamic parameters obtained in the four ways indicated above (dispersion constant, with and without dimensions, equilibrium constant obtained from the Freundlich constant and Freundlich parameter n). When the parameters are determined from the dispersion and equilibrium constants, the van’t Hoff equation is applied to determine ΔH and ΔS, and from them ΔG is obtained. When the parameter n is used, ΔG is first determined at each temperature and, with this, ΔH and ΔS are obtained. The very high value of R^2^ is noteworthy, indicating that the linearization is very good.

As can be seen in Table 3, in all cases ΔH is positive and with very similar values and higher than 100 kJ/mol, except the one determined from n whose value is somewhat lower. Such high ΔH and positive values indicate that adsorption is endothermic and with high attraction between metal and biomass, and close to chemisorption [23]. The very similar and positive ΔS values, in all cases, indicate that with increasing temperature there is a random increase in disorder at the adsorbent–solution interface and the affinity between adsorbent and adsorbate increases. Finally, high and negative ΔG values, in almost all cases, indicate that adsorption is spontaneous, and all the more so the higher the temperature. Although being less, in absolute value, than 40 kJ/mol, it is considered to be of a physical type [25].

### 3.5. Study of Biosorption Mechanisms

**Figure 4:**
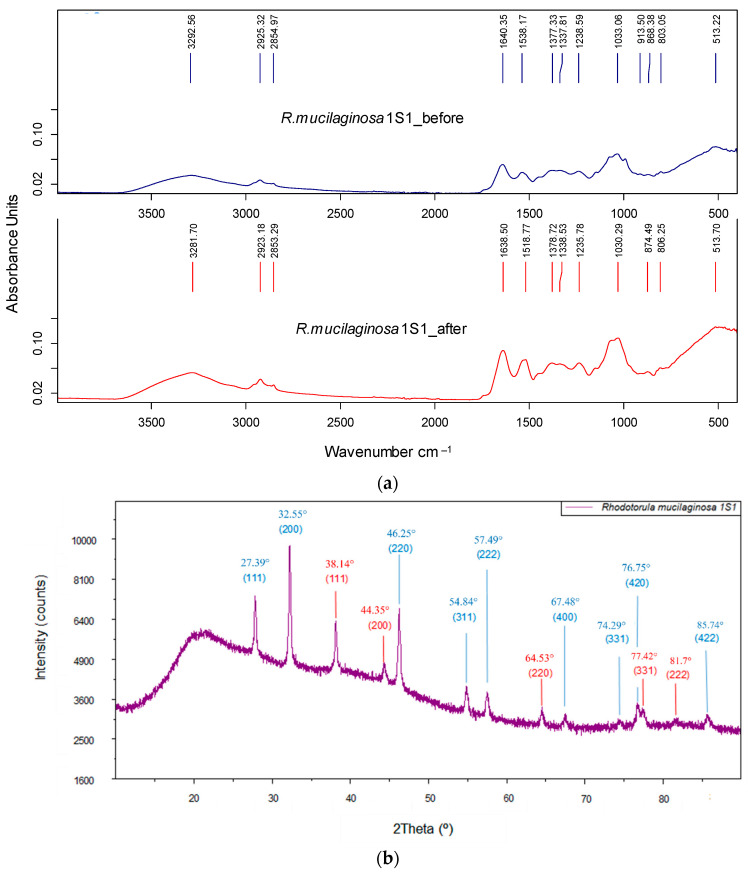
(**a**): FT-IR spectra obtained before and after the Ag(I) biosorption step for the biomass of *Rhodotorula mucilaginosa* 1S1. (**b**): XRD spectrum for biomass of *Rhodotorula mucilaginosa* 1S1 in which the sequence of peaks and the assignment of planes of the AgCl-NPs (blue) and Ag-NPs (red) can be seen.

FT-IR analysis showed a strong presence of functional groups in the biomass of *R. mucilaginosa* 1S1 and some of them were involved in the biosorption process. Figure 4a shows the spectra obtained before and after the process and in these some significant changes can be identified after silver biosorption. Three intense shifts in characteristic peaks were observed: 3293 cm^−1^ to 3282 cm^−1^ indicated the participation of amino and hydroxyl groups, 1538 cm^−1^ to 1519 cm^−1^ the participation of amide and carbonyl groups and 875 cm^−1^ to 868 cm^−1^ the participation of groups associated with stretching CH such as methyl or methylene [33,34]. The presence of two new peaks at 992 cm^−1^ and 826 cm^−1^ was also detected, the first being compatible with the participation of phosphate groups and the second compatible with C-H stretching [35]. All of the above indicated the existence of physicochemical mechanisms of surface bioadsorption and possibly bioreduction in Ag(I) mediated by hydroxyl groups that had to be confirmed later.

The XRD spectra of the biomass exposed to Ag(I) solutions are shown in Figure 4b and they clearly distinguish the characteristic peaks of two types of crystalline precipitates identified as Ag-NPs (JCPDS file 4-0783) and the crystalline form of AgCl, chlorargyrite (JCPDS file 31-1238). In both cases, the structure of the crystals was face-centered cubic (FCC). The results reveal the participation of hydroxyl groups in the reduction in Ag(I) ions and, therefore, in the formation of nanoparticles. The diffraction planes indicated in the figure are consistent with those found by other authors [36,37,38]. On the other hand, the theoretical size of the crystals obtained by the Debye–Scherrer equation from the highest intensity peak was similar for both cases with a mean diameter of 31 nm for the AgCl-NPs (peak 32.55°) and 30 nm for the Ag-NPs (peak 38.14°).

**Figure 5:**
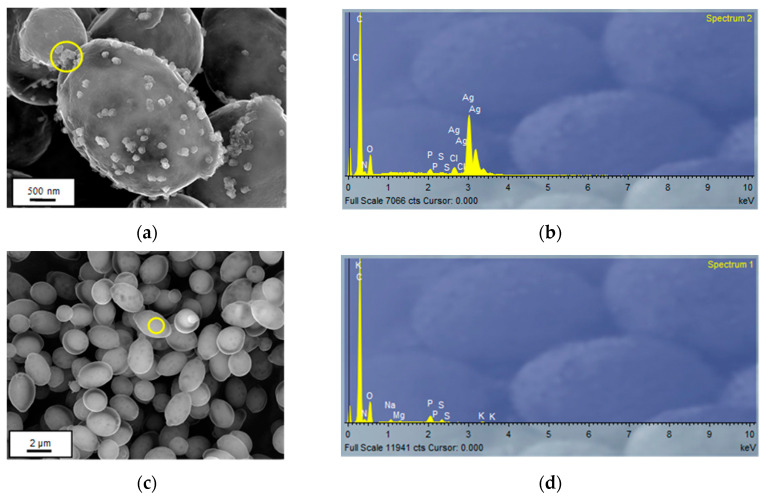

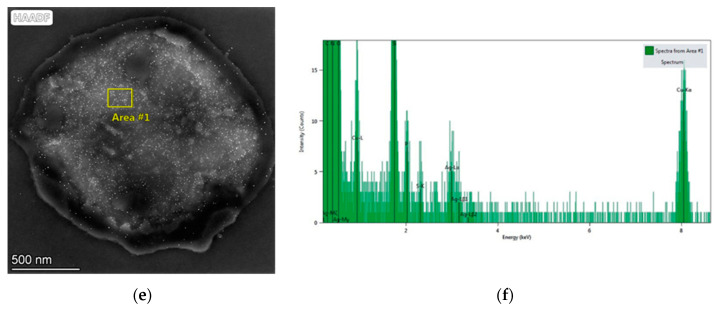
FESEM images of *Rhodotorula mucilaginosa* 1S1 cells and their corresponding EDX spectra obtained in the marked areas. (**a**,**b**) after the Ag(I) biosorption process, (**c**,**d**) before the process. (**e**): TEM-HAADF image of a *Rhodotorula mucilaginosa* 1S1 cell after the Ag(I) biosorption step. (**f**): EDX spectrum of the marked area in Figure 5e.

SEM-EDX analysis is shown in Figure 5a–d and demonstrated the existence of metal precipitates on the cell surface that form small aggregates, although elemental maps (Figure S1c) showed that silver was evenly distributed across the microbial surface. These results were consistent with those obtained in the FT-IR analysis and indicated that the surface adsorption phenomena of Ag(I) had a strong contribution to the biosorption process. At the same time, Figure S1 shows a new SEM-EDX spectrum (Figure S1b) and the elemental maps obtained after the biosorption (Figure S1c–f) on Figure S1a. When these images are analyzed, it is observed that both the EDX spectra and the elemental maps seem to indicate an association between Ag and Cl, which would support the presence of AgCl-NPs previously detected by XRD.

**Figure 6:**
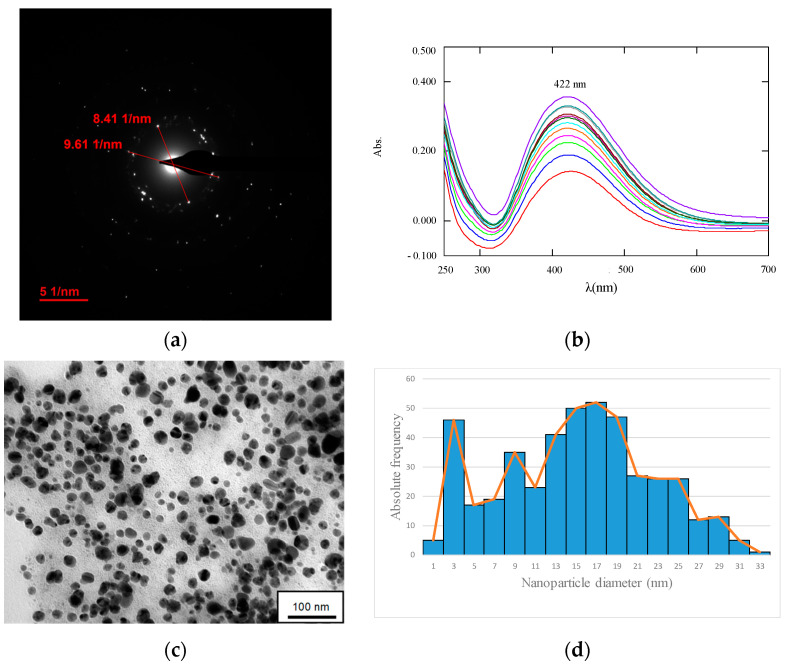

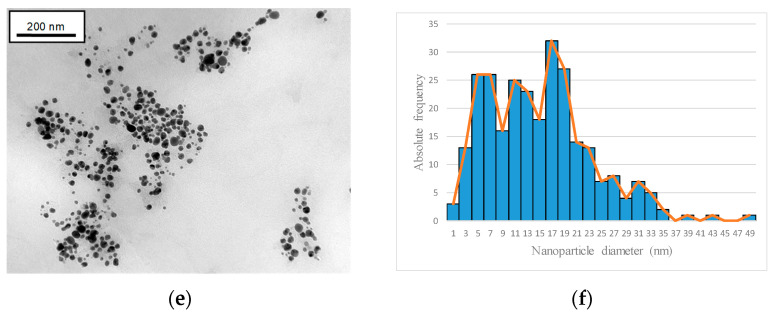
(**a**): Ag-NPs identified by electron diffraction on metallic precipitates in Figure 5e where interplanar lattice distances are shown. (**b**): UV-vis spectra obtained in the Ag-NPs synthesis assay from the cellular extract of *Rhodotorula mucilaginosa* 1S1 between 24 h (red spectrum) and 312 h (purple spectrum). TEM images of the Ag/AgCl-NPs recovered after biological synthesis from the cell extract of *Rhodotorula mucilaginosa* 1S1, (**d**,**f**) represent the histogram and frequency polygon of the size distribution obtained for images (**c**,**e**), respectively.

In parallel, TEM analysis is shown in Figure 5e,f along with Figure S2 and seems to indicate that *R. mucilaginosa* 1S1 is also capable of involving bioaccumulation mechanisms. The images show that silver was evenly distributed throughout the cytoplasm and periplasmic space, that it was not associated with chlorine and that there seemed to be some association with phosphorus (Figure S2d). When the precipitates were analyzed by electron diffraction (Figure 6a), the diameters obtained coincided with the interplanar distances of the (1,1,1) plane for the 8.41 nm diameter and of the (2,0,0) plane for the diameter 9.61 nm, and this indicated that they were Ag-NPs.

When the SEM and TEM findings are analyzed together, everything seems to indicate that the AgCl-NPs were mostly retained at the surface level and that the additional steps involved in the preparation of the TEM samples were able to remove most of the extracellular precipitates and with them, this type of nanoparticles. In short, the results allow us to suppose that *R. mucilaginosa* 1S1 involves bioaccumulation and bioadsorption mechanisms that promote the formation of Ag-NPs and AgCl-NPs, and that the former would be mainly related to cellular metabolism and therefore more present in the cytoplasm, while the latter would be associated with adsorption processes and therefore would predominate on the cell surface.

### 3.6. Synthesis of Silver Nanoparticles

Figure 6b shows the UV-vis spectra obtained during the 168 h that were necessary to complete the synthesis of Ag-NPs from the cell extract of *R. mucilaginosa* 1S1. The spectra show a characteristic peak at 422 nm that appeared from the first moment of contact and that increased in intensity as days went by. Other authors have identified similar peaks in this characteristic region of Ag-NPs, which is associated with the phenomenon known as surface plasmon resonance (SPR) [39] and causes non-absorbed light to be detected in the UV-vis spectrum. [40].

Finally, the TEM images obtained on the recovered nanoparticles after the synthesis tests are shown in Figure 6c,e and allow the identification of monodisperse spherical structures with varied sizes in which there was a predominance of the range between 12 and 20 nm (Figure 6d,f). This size is not very different from what was theoretically obtained, which was around 30 nm. 

## 4. Conclusions

The biomass of Rhodotorula mucilaginosa 1S1 turned out to be a good biosorbent of Ag(I) ions. Kinetic analysis and E_a_ values indicated that the process was dominated by adsorption of physical type. Likewise, the thermodynamic study confirmed this fact and established that it was a spontaneous process. At the same time, the model that best adjusted the equilibrium data was the Freundlich model with an R^2^ of 0.934 and a *p*-value of 0.0001 for the parameters involved and indicated that the biomass improved its performance at very dilute concentrations. The participation of amino, amide, carbonyl, hydroxyl and phosphate functional groups, as well as the corresponding mechanisms, was demonstrated by different techniques. The XRD analysis also showed the presence of crystalline precipitates of Ag/AgCl-NPs that were later also obtained in the nanoparticle synthesis assays from the cell extract, identifying characteristic UV-vis peaks for these nanoparticles with sizes between 12 nm and 20 nm. Its presence was confirmed by electron diffraction analysis, showing that the biomass of *Rhodotorula mucilaginosa* 1S1 additionally involves Ag(I) reduction mechanisms possibly mediated by hydroxyl groups identified in the FT-IR analysis. From all this, it can be deduced that this microorganism is a good Ag(I) biosorbent and at the same time is capable of synthesizing Ag/AgCl-NPs, which represents an “added value” to the biosorption process.

## Figures and Tables

**Table 1:** Kinetic parameters of Ag(I) biosorption with *Rhodotorula mucilaginosa* 1S1.

	Lagergren’s Model	Ho’s Model
a (mg/g)	−58.73	−72.03
b (K^−1^)	0.379	0.426
k_0_ (g/(mg min))	95.244	0.2028
E_a_ (J/mol)	15,229.73	8725.96
R^2^	0.900	0.957
ε (mg/g)	2.63	1.93
CV (%)	5.05	3.69
R^2^ = coefficient of determination ε = standard deviation of the model CV = coefficient of variation (relative error)		

**Table 2:** Adsorption isotherms, nonlinear fitting parameters and statistics for Ag(I) biosorption with *Rhodotorula mucilaginosa* 1S1.

Langmuir			Freundlich			Temkin			Elovich		
$q_{e}=\frac{q_{m}K_{L}C_{e}}{1+K_{L}C_{e}}$			qe=KFCe1n			$q_{e}=B_{T}\ln(K_{T}C_{e})$			Ce=qeqE1KEeqeqE		
$q_{e}=\frac{(aT+d)(pT+r)C_{e}}{1+(pT+r)C_{e}}\pm \varepsilon$			qe=(aT+d)Ce1(pT+r)±ε			$q_{e}=(aT+d)\ln\left((pT+r)C_{e}\right)\pm \varepsilon$			Ce=qe(aT+d)1(pT+r)eqe(aT+d)±ε		
		*p*-value			*p*-value			*p*-value			*p*-value
a	−1.120	0.0605	a	0.7379	<0.0001	a	−0.1116	0.2214	a	−2.058	0.0497
d	435.1	0.0178	d	−210.5	<0.0001	d	50.56	0.0704	d	700.8	0.0319
p	7.25 × 10^−4^	0.0472	p	0.1112	<0.0001	p	0.0145	0.0705	p	4.68 × 10^−4^	0.1953
r	−0.1979	0.0669	r	−30.62	<0.0001	r	−3.987	0.0905	r	−0.1233	0.2586
ε	9.5		ε	6.08		ε	6.18		ε	29.19	
CV(%)	14.11		CV(%)	8.05		CV(%)	9.77		CV(%)	16.08	
R^2^	0.776		R^2^	0.934		R^2^	0.881		R^2^	0.917	

**Table 3:** Thermodynamic parameters.

T (°C)	K_d_′ (L/g)	ΔH (kJ/mol)	ΔS, (kJ/(mol K))	ΔG (kJ/mol)
19	2.343	126.00	0.4381	−1.989
27	8.502	-	-	−5.493
37	47.316	-	-	−9.874
R^2^ = 0.999		-	-	-
-	-	-	-	-
**T (°C)**	**K_d_**	**ΔH (kJ/mol)**	**ΔS, (kJ/(mol K))**	**ΔG (kJ/mol)**
19	0.7030	126.00	0.4281	0.9359
27	2.551	-	-	−2.489
37	14.195	-	-	−6.769
R^2^ = 0.999		-	-	-
		-	-	-
**T (°C)**	**K_c_**	**ΔH (kJ/mol)**	**ΔS, (kJ/(mol K))**	**ΔG (kJ/mol)**
19	123.56	133.23	0.4976	−12.136
27	888.54	-	-	−16.117
37	3061.92	-	-	−21.093
R^2^ = 0.968		-	-	-
-	-	-	-	-
**n**	**T (°C)**	**ΔG (kJ/mol)**	**ΔH (kJ/mol)**	**ΔS, (kJ/(mol K))**
1.859	19	−4.517	83.87	0.3024
2.749	27	−6.860	-	-
3.860	37	−9.955	-	-
-	R^2^ = 1.0		-	-

## Data Availability

Not applicable.

## Supplementary Materials

The following supporting information can be downloaded at https://www.mdpi.com/article/10.3390/nano13020295/s1: Figure S1: (a): SEM image obtained after the Ag(I) biosorption step by *Rhodotorula mucilaginosa* 1S1. (b): EDX spectrum obtained in the marked region on the image (a). (c–f): Elementary maps obtained for Ag, Cl, P and S from image (a). Figure S2: TEM-HAADF image of a *Rhodotorula mucilaginosa* 1S1 cell after the Ag(I) biosorption step. (a) to (e): elemental maps for different elements. Table S1: Experimental design used in the study of Ag(I) biosorption with *Rhodotorula mucilaginosa* 1S1 and results obtained. Table S2: Experimental kinetic data of Ag(I) biosorption with *Rhodotorula mucilaginosa* 1S1. Table S3: Equilibrium experimental results on Ag(I) biosorption with *Rhodotorula mucilaginosa* 1S1.
