# Supplementary material for: Ag(I) Biosorption and Green Synthesis of Silver/Silver Chloride Nanoparticles by Rhodotorula mucilaginosa 1S1
Source: Nanomaterials (Basel). 2023 Jan 11;13(2):295. doi: 10.3390/nano13020295 (PMC9865701; doi:10.3390/nano13020295)
Supplement: Supplementary file 1 [file nanomaterials-13-00295-s001.zip › nanomaterials-2083241-supplementary.pdf]

Supplementary Materials

Ag(I) Biosorption and Green Synthesis of Silver/Silver Chloride Nanoparticles by *Rhodotorula mucilaginosa* 1S1

Antonio J. Muñoz [1,*], Francisco Espínola [1,2], Encarnación Ruiz [1,2], Manuel Moya [1,2] and Eulogio Castro [1,2]

[1] Department of Chemical, Environmental and Materials Engineering, Universidad de Jaén, Campus Las Lagunillas, 23071 Jaén, Spain
[2] Centre for Advanced Studies in Earth Sciences, Energy and Environment (CEACTEMA), Universidad de Jaén, Campus Las Lagunillas, 23071 Jaén, Spain
* Correspondence: amcobo@ujaen.es

Table S1. Experimental design used in the study of Ag(I) biosorption with *Rhodotorula mucilaginosa* 1S1 and results obtained.

A: pH	B: biosorbent dose (g/L)	q_e: biosorption capacity at equilibrium (mg/g)
5.75	0.55	46.68
4.50	0.30	69.75
5.75	0.55	46.36
5.75	0.20	-
5.75	0.55	46.57
7.00	0.80	50.86
4.50	0.80	39.64
7.00	0.30	-
5.75	0.55	47.11
5.75	0.90	58.82
5.75	0.55	46.89
7.52	0.55	56.39
3.98	0.55	29.07

Table S2. Experimental kinetic data of Ag(I) biosorption with *Rhodotorula mucilaginosa* 1S1.

19 ºC			27 ºC			37 ºC		
t (min)	q (mg/g)		t (min)	q (mg/g)		t (min)	q (mg/g)	
3	22.47	26.97	3	30.38	35.79	3	30.25	32.50
7	29.30	37.13	7	42.38	47.13	7	43.00	48.00
20	45.72	49.47	20	50.46	50.04	20	54.33	55.67
60	60.30	51.72	57	52.29	52.63	60	57.50	58.42
178	48.05	51.05	200	56.71	54.54	192	59.17	61.00
377	56.05	52.05	380	58.79	55.29	411	59.25	60.00
497	56.72	60.55	560	80.88	77.79	541	60.00	61.75
620	54.47	59.38	1290	52.96	52.63	1251	57.42	59.00
1312	56.22	55.13	1965	49.46	55.96	1471	55.17	61.83
1717	57.72	55.05	2745	55.04	59.54	1726	60.58	61.67
2057	55.13	51.38	3440	57.88	58.46	1896	58.50	59.83
2752	55.22	50.30	4235	57.88	55.84	2704	58.75	58.17

Table S3. Equilibrium experimental results on Ag(I) biosorption with *Rhodotorula mucilaginosa* 1S1*.

**19 ºC**		**27 ºC**		37 ºC	
**C_e (mg/L)**	**q_e (mg/g)**	**C_e (mg/L)**	q_e (mg/g)	C_e (mg/L)	q_e (mg/g)
16.87	40.50	17.50	38.97	16.98	41.77
27.69	37.07	28.29	38.13	28.40	41.20
37.29	39.27	38.16	38.57	37.84	45.10
63.68	49.93	65.00	48.27	66.46	43.20
99.44	50.27	100.84	54.53	99.88	61.60
134.68	60.27	135.76	70.27	138.80	70.27
164.50	63.67	165.80	70.00	168.50	68.00
260.10	114.33	269.30	85.67	275.20	81.67
354.40	130.67	373.90	72.67	367.60	114.00
459.00	124.33	494.00	61.67	485.40	110.67
15.76	44.20	16.94	40.83	17.04	41.57
27.89	36.40	28.50	37.43	28.45	41.03
37.44	38.77	37.02	42.37	37.84	45.10
63.02	52.13	64.34	50.47	65.66	45.87
99.36	50.53	98.40	62.67	101.64	55.73
134.52	60.80	138.92	59.73	138.64	70.80
155.60	93.33	165.70	70.33	165.60	77.67
259.20	117.33	270.90	80.33	276.20	78.33
360.00	112.00	368.20	91.67	375.60	87.33
468.30	93.33	481.20	104.33	477.50	137.00

* The experimental series was carried out in duplicate.

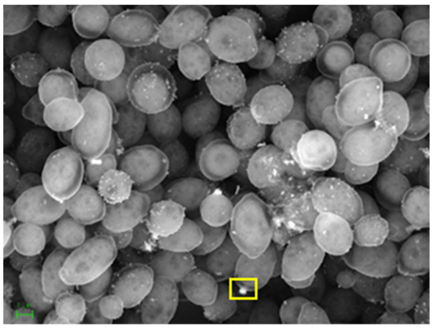

(a)

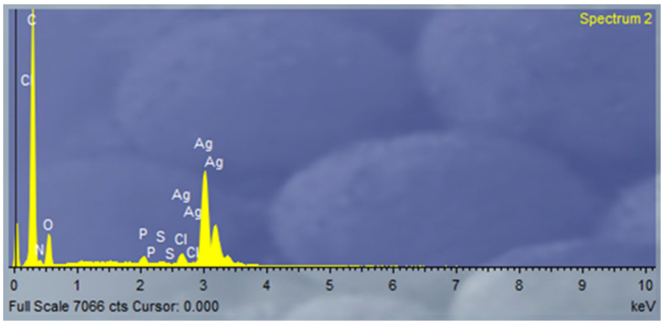

(b)

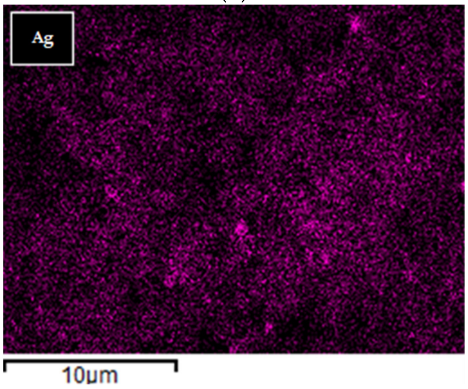

(c)

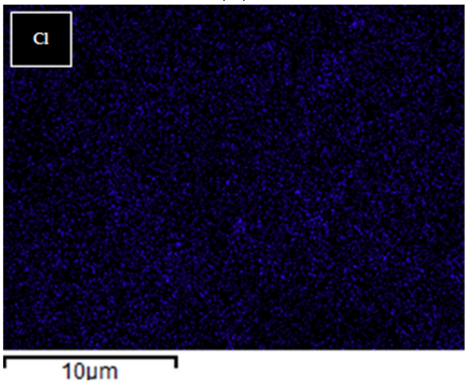

(d)

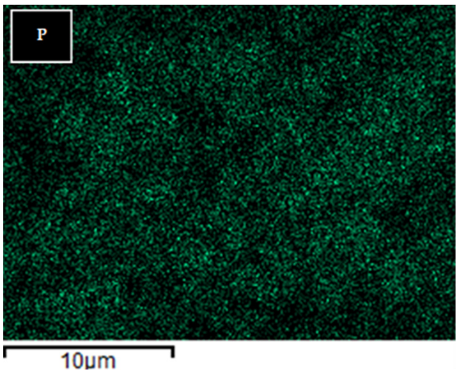

(**e**)

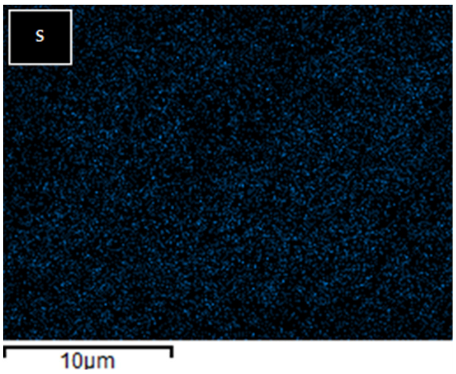

(**f**)

Figure S1. (a): SEM image obtained after the Ag(I) biosorption step by *Rhodotorula mucilaginosa* 1S1. (b): EDX spectrum obtained in the marked region on the image (a). (c) to (f): Elementary maps obtained for Ag, Cl, P and S from image (a).

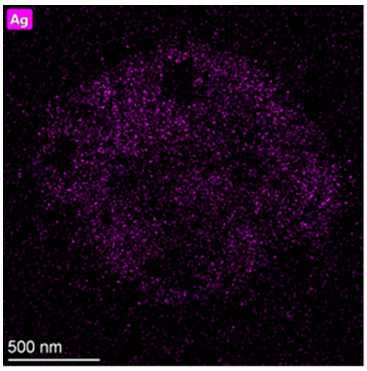

(**a**)

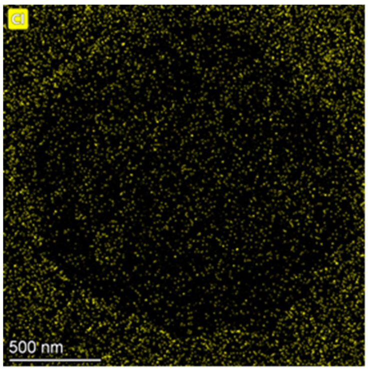

(**b**)

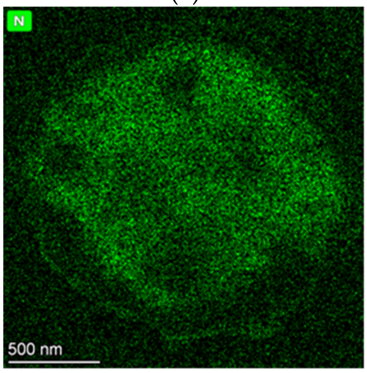

(**c**)

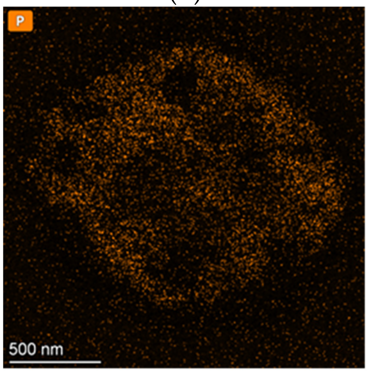

(**d**)

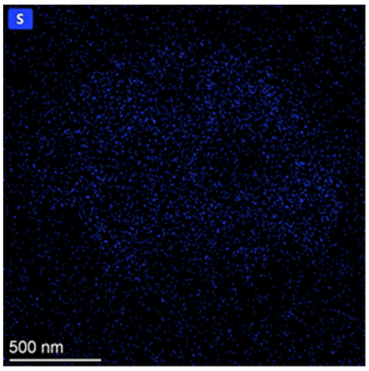

(**e**)

Figure S2. TEM-HAADF image of a *Rhodotorula mucilaginosa* 1S1 cell after the Ag(I) biosorption step. (a) to (e): elemental maps for different elements.
